# A Two-Stage De-Identification Process for Privacy-Preserving Medical Image Analysis

**DOI:** 10.3390/healthcare10050755

**Published:** 2022-04-19

**Authors:** Arsalan Shahid, Mehran H. Bazargani, Paul Banahan, Brian Mac Namee, Tahar Kechadi, Ceara Treacy, Gilbert Regan, Peter MacMahon

**Affiliations:** 1 School of Computer Science, University College Dublin, D04 V1W8 Dublin, Ireland; mehran.hosseinzadehbazargani@ucd.ie (M.H.B.); brian.macnamee@ucd.ie (B.M.N.); tahar.kechadi@ucd.ie (T.K.); 2 Department of Radiology, Mater Misericordiae University Hospital, D07 R2WY Dublin, Ireland; paulbanahan@eril.ie (P.B.); pmacmahon@mater.ie (P.M.); 3 Regulated Software Research Centre, Dundalk Institute of Technology, A91 K584 Dundalk, Ireland; ceara.treacy@dkit.ie (C.T.); gilbert.regan@dkit.ie (G.R.)

**Keywords:** privacy preservation, de-identification, DICOM, medical image analytics

## Abstract

Identification and re-identification are two major security and privacy threats to medical imaging data. De-identification in DICOM medical data is essential to preserve the privacy of patients’ Personally Identifiable Information (PII) and requires a systematic approach. However, there is a lack of sufficient detail regarding the de-identification process of DICOM attributes, for example, what needs to be considered before removing a DICOM attribute. In this paper, we first highlight and review the key challenges in the medical image data de-identification process. In this paper, we develop a two-stage de-identification process for CT scan images available in DICOM file format. In the first stage of the de-identification process, the patient’s PII—including name, date of birth, etc., are removed at the hospital facility using the export process available in their Picture Archiving and Communication System (PACS). The second stage employs the proposed DICOM de-identification tool for an exhaustive attribute-level investigation to further de-identify and ensure that all PII has been removed. Finally, we provide a roadmap for future considerations to build a semi-automated or automated tool for the DICOM datasets de-identification.

## 1. Introduction

Machine Learning (ML) can be described as a disruptive technology that uses algorithms to dissect complex datasets. In the world of medical image analysis, ML is increasingly used to facilitate the detection and quantification of a wide array of clinical conditions [1]. Furthermore, ML is also used to analyse and extract meaningful knowledge from unstructured clinical datasets. Additionally, it may be a valuable ally for radiologists and pathologists looking to accelerate their productivity and potentially improve their accuracy [2].

A report from the Emergen Group (Medical Image Analytics Market Size, https://www.emergenresearch.com/industry-report/medical-image-analytics-market, accessed on: 4 April 2022) indicates that the Medical Image Analytics market will be worth 4.66 billion USD by 2027. This is mainly due to: an increase in evidence of their benefit and the prevalence of chronic diseases; unusual incidents in the pharmaceutical industry; and a rise in government and private financing. However, there are a number of constraints to the medical image analytics market. These include the high cost of advanced image systems such as Picture Archiving and Communication Systems (PACS) and increasing concerns around data protection.

Exchange of medical images, specifically in today’s environment of cloud computing and over public networks, exposes them to different types of security [3] and privacy [4] threats. This is vastly different from the closed networks, which the healthcare domain has traditionally been based in [5]. This raises concerns about compromised patient privacy and the security of the data associated with the medical images. Ensuring the security and privacy of personal data, including medical data, is a major factor and has been mandated by regulation. The key EU regulation for data protection is the General Data Protection Regulation (GDPR) [6]. The data protection principles of the GDPR regulation are provided in Article 25, which is one of the most critical pieces mandated by the regulation. Article 25 relates to establishing all necessary measures to protect personal data [7]. Article 25 requires “data protection by design and by default” (General Data Protection Regulation (GDPR), Article 25: https://gdpr.eu/article-25-data-protection-by-design/, accessed on: 4 April 2022). This article requires that data protection is built into the core of technical products and implemented into the design of any system processing personal data [8]. This requirement means that both security and privacy are employed from the beginning of system development [8].

The Medical Imaging Ireland (Med-I) project (Funded. by Enterprise Ireland Disruptive Technologies Innovation Fund) is devoted to medical image analysis. The main objective of Med-I is to deliver a platform offering and enabling technologies that can host, manage, process and analyse digital medical images. While Med-I collects partially de-identified data exported from major healthcare provider’s Picture Archiving and Communication Systems (PACS), it is necessary to further de-identify the data to ensure privacy. An issue with de-identification is the lack of sufficient detail concerning the de-identification process of the DICOM attributes. Therefore, Med-I proposes a two-stage process that provides a semi-automated de-identification of data, which includes the best practices for de-identifying DICOM headers. A demonstration of how these practices are employed in the two-stage process developed by the Med-I project is also included.

Using ML for medical imaging analytics requires data-driven platforms to process highly sensitive medical data. As noted, the preservation of the privacy of these data is mandated by regulation. In addition, processing this type of data requires care and consideration of ethical and legal concerns. The objective of the Med-I project is to design a data-driven platform in such a way that the issues of privacy and ethics, as well as the big data challenges, are addressed in an efficient manner.

Digital Imaging and Communication On Medicine (DICOM) is an international standard in the medical domain and is supported by all the vendors of clinical imaging systems [9]. A DICOM file consists of the actual image, accompanied by some metadata, as part of its header. These headers can include information (attributes) such as; name, phone number, home address, email address, social security number, etc. One way of concealing the identities that are present in the data is to remove all header information, which can be used to identify any of the individuals. This task is usually called de-identification. In most of the data analysis processes, these attributes are deemed not to be important and, therefore, are removed. However, they can be used during the data preparation to generate other dimensions or fill in missing values, etc. Moreover, depending on the application, some attributes may need to be de-identified to preserve a data subject’s identity. Usually, when the data are de-identified, a new identifier is used so that the new derived data are not linked to the original data.

In many cases, de-identified data are enough to reach an acceptable level of privacy protection. The data are deemed anonymous and can be shared within the community. However, for highly sensitive data or data that requires a higher level of data privacy protection, the de-identification process cannot be validated without the integration of other protocols and techniques. The study presented by Sweeney [10] confirmed that the validation process is extremely important before sharing any de-identified data sets. Furthermore, the de-identification process comes with many challenges that need to be addressed before any data collection or analysis.

In this paper, we present the de-identification techniques for digital medical images that require regulatory mandated confirmation that the data are de-identified and in line with consent for its use. The paper presents the challenges and a validation process for meeting these requirements. We proposed a two-stage de-identification process that performs a detailed attribute-level exploration for removing the Personal Identifiable Information (PII) and Protected Health Information (PHI) in medical image datasets.

The rest of this paper is organised as follows: Section 2 presents the literature review with DICOM de-identification techniques and challenges. In Section 3, we first highlight the specifics of DICOM image processing followed by the important considerations towards developing a DICOM de-identification process. Section 4 presents the proposed two-staged DICOM de-identification process. In Section 5, we discuss some learned lessons and provide a road-map for future considerations. Finally, Section 6 concludes the paper.

## 2. Related Work

The de-identification and data curation processes are very complex and time-consuming. Furthermore, they require rigorous evaluation before they can be deployed to process real-world data. The de-identification methods can be divided into two main categories: (1) De-identification of the DICOM header and (2) De-identification of PII and PHI embedded in the medical images at the pixel level.

### 2.1. DICOM Header De-Identification Techniques

In Muschelli et al. [11], the DicomCleaner tools are used for CT data processing, including a de-identification tool to extract DICOM header and PHI information. The author recommended de-identifying the data using a clinical trial processor and converting it to Neuroimaging Informatics Technology Initiative (NIfTI) format. However, the in-depth attribute-level process to deal with sensitive metadata is not discussed. Moore et al. [12] proposed a list of operations for DICOM de-identification for various attributes including replacing, removing, cleaning, etc. The authors highlighted that, as the DICOM public elements have been defined in the DICOM standard, there are many self-defined private elements, and they are not standard. Common practices to deal with private attributes include deleting them completely with a risk of losing the scientific value of the data.

In [13], a web-based software tool was developed to extract, de-identify, and edit DICOM files. MIRC DICOM anonymiser, which is an open-source package, is integrated into the developed Teaching File Transfer Tool (TFTT). The de-identification process complies with Health Insurance Portability and Accountability Act (HIPAA), that is, all HIPPA direct identifiers are removed from the DICOM headers. It is worth mentioning that the TFTT software supports manual pixel de-identification operations, such as cropping and blackout. However, the de-identification process lacks a detailed description. For instance, it is mentioned that the *SOP instance UID* attribute is hashed; however, details are missing as to how the necessary consistency amongst UIDs, dictated by the DICOM standard, is preserved. As another example, while removing a given attribute, it is not clear whether the attribute type is consulted with the DICOM standard (to confirm whether the action is compliant with the DICOM standard). Similarly in [14], a list of Protected Health Information (PHI) DICOM attributes is provided, which consists of the minimum set of attributes for de-identification recommended by the DICOM standard, to be either removed or replaced. Unfortunately, the de-identification process lacks details. For instance, it has been mentioned that *Instance UIDs* have been replaced consistently; however, the methods and conditions of keeping this consistency have not been discussed.

Haak et al. [15] proposed a web-based platform for capturing and managing DICOM data for multi-centre trials. Data privacy measures include automatic de-identification and re-labelling techniques that remove the obvious PHI from the DICOM headers such as patient name, birth date, birth time, sex, etc. However, the authors did not mention the process of dealing with *UIDs* to preserve privacy, and DICOM standards have not been consulted. Aryanto et al. [16] presented a comparative study of non-commercial DICOM de-identification tools for their performance in removing PHI. The authors discussed the existing DICOM de-identification techniques, including the removal of the DICOM headers with PHI and the pseudonymization method that replaces the identifiable attributes within a separate data record. These records can be used to track the patients. The authors listed several DICOM attributes that need to be de-identified but did not include the UID attributes in the evaluating process. On average, the success rate of privacy preservation for DICOM de-identification tools is 48%. Some DICOM tools including DICOM Library (https://www.dicomlibrary.com/, accessed on: 4 April 2022) and CTP outperform others with over 90% success rate.

Saad et al. [17] proposed an architecture for pseudonymising the metadata of the DICOM real-time vide generated inside the medical operating rooms. The proposed methodology includes the pseudonymisation of DICOM data during live video streaming of surgery for it to be shared outside hospital premises. However, the de-identified data can be re-identified using a stored record of the original DICOM metadata. Furthermore, the authors did neither discuss nor provide details on the attribute-level operations to de-identify the metadata.

Beasley et al. [18] presented a secure-DICOM-uploader to de-identify and share medical data outside hospital premises. The authors stated that private DICOM headers are programmatically removed but did not provide fine-grained details on the overall de-identification process.

Thomas et al. [19] presented a platform for DICOM image analytics and archive (DIANA) containing a module for removal of PHI from the DICOM header. Although the authors claimed that they evaluated their platform for the PHI removal, the authors did not provide details of methodologies employed in DIANA for dealing with the DICOM header to ensure privacy preservation. Similarly, there are a number of works that claim to tackle the de-identification of DICOM header attributes but lack the fine-grained details of the process to ensure patients’ privacy [20,21,22].

Table 1 compares the recent works on DICOM header de-identification in terms of fine-grained de-identification details, conforming to DICOM standards, validation with expert radiologists, open-source availability, and deployment in the production environment. It can be seen that the proposed method ticks all the boxes in comparison with the state-of-the-art in all aspects.

### 2.2. Pixel-Level De-Identification in DICOM Images

De-identification of burned-in annotations and PHI embedded in DICOM pixel data is a critical task for privacy preservation. Robinson [23] discussed the need for preserving the privacy of patients in DICOM images in addition to the PII in the headers.

Monteiro et al. [24] presented a mechanism for HIPAA compliant ultrasound medical image de-identification that is exposed as a REST API and a Software-as-a-Service (SaaS) based cloud solution. The proposed solution comprises Machine Learning (ML) models to build an automated process for PII identification embedded in the pixel data. The character recognition tasks are performed using Convolution Neural Networks (CNNs) to achieve a de-identification accuracy of 89.02%. Vcelak et al. [25] proposed an adaptive-iterative algorithm to classify the images with and without burned-in pixel data in DICOM. Various image transformations were applied, including noise filters and optical character recognition. The evaluation of the proposed method showed a false positive rate of up to 4% for identifying PHI with a recall score of 94.85%. Rutherford et al. [26] collected an extensive dataset (a total of 1693 CT, MRI, and other formats of medical images selected from the Cancer Imaging Archive (Cancer Imaging Archive, https://www.cancerimagingarchive.net/, accessed on: 4 April 2022), i.e., TCIA) to evaluate the performance of the de-identification algorithms in preserving the privacy of patients.

Although ML methods can be effectively used to automate the de-identification process in pixel data, the proposed process relies on complex and computationally expensive operations, such as image pre-processing, object detection, and image resolution. The complexity of the DICOM image processing is due to the large size of datasets. For example, a typical CT DICOM scan consists of up to 200–500 slices/images, with each slice/image composed of 512 × 512 pixels. Therefore, building a medical imaging analytics platform and establishing DICOM data pre-processing pipelines is not a trivial task. Furthermore, the ML processing needs advanced classifier models to deal with different modalities to obtain the desired results.

González et al. [27] presented the requirements of the DICOM de-identification process and developed a flexible toolkit by embedding mechanisms to de-identify images from multiple sources. The authors reviewed the DICOM de-identification tools and found that the existing tools do not satisfy essential privacy preservation criteria, such as handling burned-in annotations and supporting attribute-level confidentiality. The authors proposed a PrivacyGuard toolkit, a Java-based open-source application, to ensure the enforcement of privacy policies. However, the toolkit integration within modern cloud-based infrastructures is not a straightforward task. Furthermore, the authors did not give the attribute-level handling details and toolkit evaluation for real-world datasets.

To de-identify the pixel data containing the burned-in text, Clunie et al. [28] presented an open-source selective redaction tool to identify the image blocks in JPEG that contain text with PHI, and demonstrated its applicability to a use-case with multi-frame colour ultrasound images. The authors discussed how the process can be applied to JPEG images individually or the streams of JPEG combined into a DICOM format. Some limitations of the process include processing the entire blocks in redacted regions instead of redacting only the sub-parts of the image blocks.

Li et al. [29] presented a tool for DICOM brain image de-identification that removes PII in the form of facial features, such as nose, lips, ears, etc., without disturbing the quality of data and by preserving the brain tissues. The presented tool performs three functions; image defacing, de-identification and data sharing. Noise reduction, locating the region of interest, and image cropping are performed to deface the medical images. The de-identification process includes the data items selection and replacing them with randomly created strings. Finally, the data are shared on remote FTP servers. The tool provides a one-stop service to de-identify DICOM images with facial features through a consistent three-phase methodology.

Kundu et al. [30] presented a standalone de-identification tool for clinical and annotated medical imaging data, including Radiotherapy Structure Set (RTSTRUCT), Radiotherapy Plan (RTPLAN), and Radiotherapy Dose (RTDOSE). The clinical data includes; text-based notes reporting diagnosis, stages, outcomes, and treatment plans for patients and the imaging data include diagnostic images. The proposed process is to keep the reference of the original data in an encrypted form that can be used for re-identification. The de-identification system is installed in the hospital premises under the protected network to reduce the risk of malware attacks.

To summarise, privacy preservation of PII and PHI embedded in DICOM headers is a significant growing concern due to the sensitive nature of the data and regulatory requirements. The breach of PII and PHI embedded in DICOM headers can result in substantial GDPR fines, damage to an organisation’s reputation and loss of trust from the data subjects. The main issue with the DICOM header de-identification in the literature is the lack of sufficient detail regarding the de-identification process of the attributes. For instance, what needs to be considered before deciding to remove a DICOM attribute or replace its value to make sure the de-identified DICOM file still complies with the DICOM standard? The PII and PHI attributes must be identified and removed without degrading the integrity of DICOM standards. In the current dataset, even though there are no PHI and text annotations embedded at pixel-level in the DICOM scans, a well-defined and detailed methodology of dealing with sensitive information embedded in the DICOM headers is employed, which complies with the DICOM standard.

## 3. Material and Methods

Unlike other types of data, the format of imaging data is fundamental as it not only describes the image data at a pixel level but also stores additional relevant information related to the image. Different formats describe digital images differently, based on some key objectives, which are usually related to a given application domain. In medical imaging data, some formats have emerged, including DICOM, NIfTI, MINC, and ANALYZE.

- *DICOM (Digital Imaging and Communication On Medicine)*: is an international standard in medical domain and covers all imaging modalities and organs [9]. DICOM is supported by almost all vendors of clinical imaging systems.
- *ANALYZE*: This format is widely used in the functional neuroimaging field [31]. Each data item consists of two files: (1) a file with contains the actual data in a binary format with the filename extension *.img*, and (2) a header file with filename extension *.hdr* and it holds information about the data, such as voxel size and the number of voxels in each dimension. This format is progressively replaced by NIfTI.
- *NIfTI (Neuroimaging Informatics Technology Initiative)*: This is a format for neuroimaging developed in the early 2000s by the DFWG (Data Format Working Group) to improve the ANALYZE format [32]. The initial version of NIfTI, that is NIfTI-1, combines header and image sections. Extensions of NIfTI provide flexibility to store additional information such as experimental design and data acquisition details. More recently, the Brain Imaging Data Structure (BIDS) (Brain Imaging Data Structure, https://bids.neuroimaging.io/, accessed on: 4 April 2022) format is rapidly replacing NIfTI.
- *MINC (Medical Imaging NetCDF)*: This format was designed for medical imaging data and built upon the NetCDF (Network Common Data Format) standard [33]. MINC provides a modality-neutral way to store medical images alongside text data [34].

In this paper, the medical images are collected from CT scanners and are stored in the DICOM format containing various PII.

### 3.1. DICOM Image Processing

**Figure 1:**
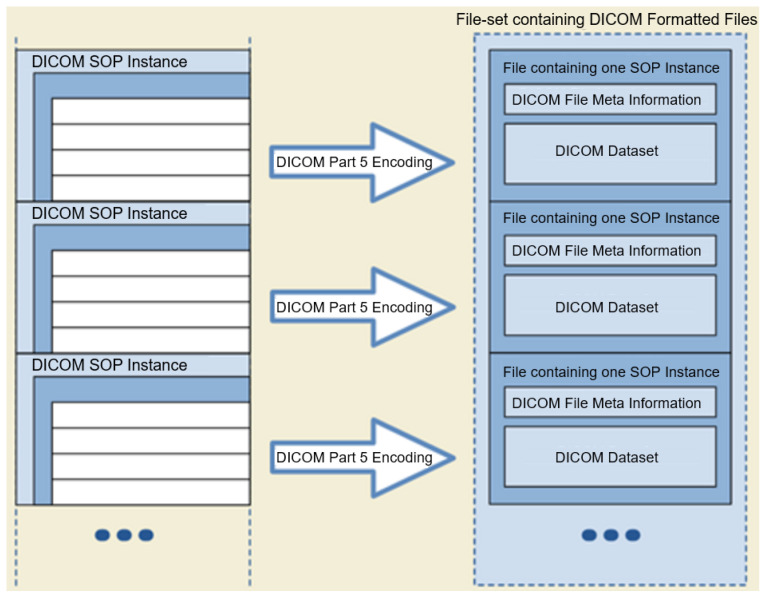
DICOM File-set and File Format (adopted from the DICOM standard, Chapter 7).

The DICOM format (Extensive documentation on the DICOM standard is provided at https://www.dicomstandard.org/, accessed on: 4 April 2022) is a globally accepted medical imaging file format. It complies with a set of protocols for maintaining the accuracy and integrity of medical images [9]. The DICOM file format was initially designed by the American College of Radiology (ACR) and the National Electrical Manufacturers Association (NEMA) to view and share medical images of different types; CT scans, MRIs, and ultrasound. The first version of the format was released in 1985, followed by the second version in 1988, which became popular among medical device vendors. The third iteration was released in 1993 and became the backbone of all the later DICOM versions until now. The DICOM format provides a means for encapsulating images with their metadata in a DICOM file. Figure 1 shows the DICOM format including the File Meta Information (DICOM file format: http://dicom.nema.org/medical/dicom/current/output/chtml/part10/chapter_7.html, accessed on: 4 April 2022).

The DICOM images, in addition to the actual image-related data (including the actual pixel values of the image), contain a header often referred to as metadata. The metadata may store PII, such as name, ID, sex, and birth date, or crucial acquisition data information, such as the compression method used or details about the type of equipment used to produce the image.

Each attribute (DICOM data element, https://dicom.nema.org/dicom/2013/output/chtml/part05/chapter_7.html, accessed on: 4 April 2022) (i.e., element) in a DICOM file consists of up to four main segments: Tag, Value Representation (VR), Value Length, and Value Field. Here is a brief description of each segment:

- *Tag:* Each DICOM element in a DICOM file has a tag, which uniquely defines that element. A DICOM tag consists of 2 main parts: (1) Group number (2) Element number. For instance, an element with the Tag (0028, 0010) belongs to the Image group 0028 (i.e., attributes that describe the image and its properties). The element number 0010 corresponds to the number of rows in the image. Similarly, under the same group, element number 0011 refers to the number of columns in an image. In the case of the dataset used in this paper, both the number of rows and columns are 512.
- *Value Representation (VR):* A VR is a two-character code that defines an element’s type. There are a variety of data types in DICOM. For instance, *UI* refers to a Unique Identifier data type, and *SH* refers to a *Short String* data type.
- *Value Length:* This shows the length of a DICOM element. For example, the length of the value of the *Patient Sex* element (i.e., ‘F’ for female, ‘M’ for male, and ‘O’ for other) is 2 (note the trailing space which is mandated by the VR for this element).
- *Value Field:* This holds the actual value of the element. Values with VRs, consisted of character strings (except for the UI VRs), need to be padded with a space character when necessary to achieve even length. On the other hand, values with a VR of UI should be padded with a single trailing NULL character when necessary to achieve even length. Finally, values with a VR of OB (i.e., Other Byte String) also need to be padded with a trailing NULL to ensure even length. During de-identification or any type of value manipulation, these rules need to be followed.

Each DICOM attribute is also associated with a type called the *Data Element Type*. These element types should be considered, before de-identifying a given attribute in order to not violate the DICOM standard. In particular, these data element types are as follows:

- *Type 1:* Attributes under this category must be present and have a valid value (depending on the VR of the element).
- *Type 2:* Attributes under this category must be present, however, they may contain the value of “unknown”, or simply a zero-length value (unlike Type 1 attributes).
- *Type 3:* Attributes under this type are optional. This means that they may or may not be included in the DICOM header and if present, their value could be of zero length. Removing such attributes is not a violation of the DICOM standard.
- *Type 1C:* The letter C stands for Conditional. Attributes under this category cannot be simply removed as their existence is conditional. If the condition is met, then the attribute shall be treated as a Type 1 attribute (i.e., their existence is required and cannot have a zero-length value). If the condition is not met, these attributes will not be included in the dataset. They cannot be simply de-identified without investigating the condition, since it is indeed a DICOM protocol violation if the specified conditions are met and a Type 1C attribute is not included.
- *Type 2C:* Similar to Type 1C attributes, the existence of the attributes under this category is conditional. If the specified conditions are met, then the attribute is treated as a Type 2 attribute. If the specified condition is not met, the attribute may not be included in the dataset. Such an attribute cannot be simply de-identified without investigating the required conditions that dictate their existence, and it is indeed a DICOM protocol violation if the specified conditions are met and a Type 2C attribute is not included due to removal.

In addition, it is worth noting that there is a DICOM data model, which dictates certain relationships between individual images. It is indeed the definition of a relational database, which defines Information Entities (IEs): Patient, Study, Series, Image, and their relationship to each other. The Patient ID is at the top of the hierarchy. For a given Patient ID, multiple studies can take place, each of which has a unique Study Instance UID. Each study can then have several series identified by Series Instance UIDs. Finally, each series can have multiple DICOM images, where each slice has an SOP Instance UID. Usually, an Instance UID is a unique identifier assigned to a DICOM instance/image. For example, Study Instance UID identifies the study to which the DICOM image belongs, and Series Instance UID identifies, within a particular study, to which series the DICOM image belongs.

### 3.2. DICOM De-Identification Considerations

A de-identification process must look into two components of a DICOM image that may hold PII. The first component is the DICOM header. The DICOM de-identification process primarily removes or overwrites the patients’ PIIs, including their names, date of birth, gender, address, etc., from the DICOM header [35]. Secondly, in some cases, the pixel/voxel data of the images also requires de-identification, especially in the case of CT scans. Head images and face regions are de-identified because they may contain either burned-in PHI, data annotations or exposed physical body features that can disclose a patient’s identity using image identification exploits.

A DICOM file contains sensitive attributes. These can disguise the identity of a patient. However, one needs to consider the type (i.e., 1, 1C, 2, 2C, or 3) of a given DICOM attribute before deciding on the method of de-identification. In general, for a given DICOM attribute, there are three possible de-identification operations:

- *Clear:* The attribute will remain; however, its value will be cleared to an empty string of length 0 (the VR of the attribute must be consulted).
- *Overwrite:* The attribute will remain; however, its value will be overwritten with a word such as ‘CONFIDENTIAL’ or a randomly assigned value (the VR of the attribute must be consulted).
- *Remove:* The entire attribute will be removed from the DICOM file (the attribute type must be consulted).

Depending on the type of attributes, certain de-identification actions are allowed. For instance, removing a Type 1 attribute is a definite violation of the DICOM standard, whereas, removing a Type 3 attribute is allowed. The following are the sensitive attribute categories that need to be carefully handled in the de-identification process:

- *UID/ID:* DICOM uses UIDs to make possible the communication between multiple application entities over a PACS system. The DICOM standard uses certain UID’s so that a sender and a receiver node in a PACS system can encode/decode the transferred DICOM files (i.e., they can talk DICOM). For instance, the sender can use a certain value for the Transfer Syntax UID to inform the receiver of the compression algorithm used to compress the data by the sender, so that the receiver can successfully decompress the data upon receiving it. Such UIDs take publicly available standard values. There is a variety of services available in DICOM. For instance, the standard SOP Classes and their UID classes for Storage services can be found in section B.5 of the DICOM standard (Standard SOP Classes, https://dicom.nema.org/dicom/2013/output/chtml/part04/sect_B.5.html, accessed on: 4 April 2022). As mentioned in the previous section, some of the UIDs and IDs are also used to maintain links between the patient, study, series, and DICOM instances.
  Among all of the different UIDs in the collected DICOM dataset, it has been confirmed that it is only the instance UIDs that bear a potential risk of disclosing the patient’s identity. Because other types of UID within this dataset (e.g., SOP Class UIDs, Transfer Syntax UID, Irradiation Event UID, Frame of Reference UID, etc.) are either about the method of data transmission over a PACS network, method of data storage, or simply keeping track of the frame of reference in a given series. For instance, the SOP Class UID definition (globally known and unique) contains the rules and semantics which may restrict the use of certain services for the current data at hand. For example, the SOP Class UID of 1.2.840.10008.5.1.4.1.1.2 refers to the CT Image Storage service, and the SOP Class UID of 1.2.840.10008.1.1 refers to Verification SOP Class (i.e., verification of basic connectivity between a sender and receiver over a PACS network). For a given DICOM file, these Class UIDs have nothing to do with the data within the file and only describe the type of service that is required for that file.
  While a Class UID is similar to a governing template with no actual patient-related data, Instance UIDs have a direct connection with the actual data within a DICOM file. For instance, for a given DICOM file, Study Instance UID and Series Instance UID can directly and uniquely point to which exact study, and within that study, to which series that particular DICOM file belongs.
  Patient ID and Study ID need to be de-identified to protect the identity of the patient. It is important to note that in the dataset used in this paper, it was found that Class UIDs (as opposed to Instance UIDs) were not de-identified by the export process withing the hospital since they do not disclose anything about the patient’s identity. On the other hand, all Instance UIDs were de-identified by the export process.
- *Dates/Times:* There are many date and time attributes in a DICOM header and it is customary to replace their values with some randomly generated date and time strings.
- *Sensitive names:* There are names such as; Station Name, Patient Name, Physician’s Name, Radiologist’s Name, Institution Name/Address, that require de-identification.
- *Sensitive numbers:* There are certain numbers that one might need to be de-identified. One such attribute, Accession Number, is a RIS (Radiological Information System) generated number that identifies the order for the Study. This value could be used to reconstruct the identity of the patient [16].

## 4. De-Idenfication Process

This section first reviews the challenges of medical imaging data de-identification. Furthermore, a two-stage process for DICOM header de-identification is presented. In the first stage of the process, the DICOM header attributes are de-identified at the data collection premise, i.e., Ireland’s National Integrated Medical Imaging System (NIMIS) through their export process, which is also called the de-identification at the source. NIMIS is a PACS network employed in Irish hospitals, initiated in 2008 by Health Service Executive (HSE). In the second stage of the de-identification process, a detailed attribute-level exploration is performed to investigate the remaining PII. We also developed an open-source tool (the source code of de-identification tool employed in the second stage of the de-identification process and is publicly available at: https://csgitlab.ucd.ie/mldawn/dicom_de_identifier_public, accessed on 4 April 2022). This tool was developed to automate the de-identification process in compliance with DICOM standards [36]. The challenges for de-identification of medical datasets include the following:

- Different regulations and conditions appear in different authorities such as GDPR and HIPAA, complicating the development of international standards for de-identification in medical imaging data [37].
- De-identification techniques are usually employed in the preparation stages for data transfer or sharing [38]. In cases where the patient withdraws their consent, it detaches data governance from data ownership (hindering the right to be forgotten, GDPR article 17 [39]). In addition to this, if the legislation changes, the new data that will be generated from the original data should *not* include those patients data. In this case, the sharing protocol should be clear of ambiguity and defined to satisfy the data owners requirements.
- Requirements of the de-identification process vary according to the type of datasets [40]. For example, it is harder to link a radiographic image of a section of the leg back to an individual, compared to a computed tomography scan of their head, where the contours of the face can be reconstructed directly from the image.

As mentioned above, the proposed de-identification process consists of two stages: (1) De-identification of DICOM data at source, which uses the capabilities of the PACS system at the clinical site; and (2) de-identification after extraction to ensure that the data are fully de-identified.

### 4.1. Stage 1: De-Identification at Source (NIMIS)

**Figure 2:**
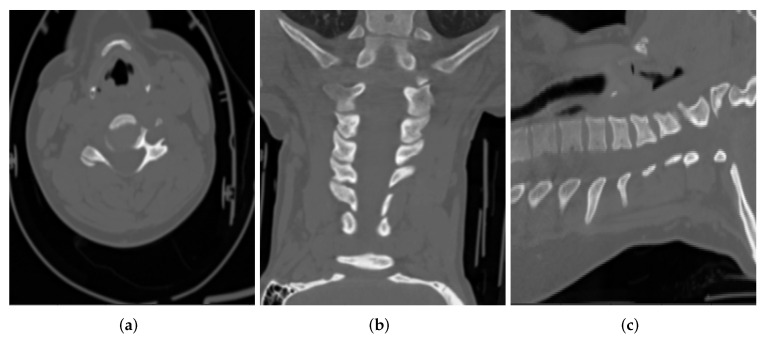
Sample CT images from (**a**) Axial, (**b**) Coronal and (**c**) Sagittal views for a patient.

The export process at the source does a first round of de-identification on the DICOM files. These files are then put through a second round of de-identification/post-processing for further validation. As an example of DICOM CT images, de-identified at the source, Figure 2 shows DICOM CT images belonging to a 65 year old female patient, in three main views (i.e., axial, coronal, sagittal):

The data directory of the partially de-identified DICOM CT-scan images received from the source has the following structure:

The directory names include an indicator of the sex of the patient (F for female in the example above) and the patients’ age range (25–30 in the example above). For example, a directory name of CASE10 F 25_30/, points to the images of the 10th patient case, who is a female and in the 25–30 age range. The research team decided that the age ranges will always follow this pattern: 15–20, 20–25, 25–30, through to 100–105 years. The age range decision was implemented to further preserve PII privacy and preserve unlinkability.

The export process at the source takes care of the de-identification to a decent degree, where *some* of the critical attributes such as patient’s name, ID, birth date, sex, etc. have been de-identified. Below is a summary of this process:

- The following Instance UIDs are changed while preserving the relationship in the DICOM data model:
  – *Media Storage SOP Instance UID:* Overwritten
  – *SOP Instance UID:* Overwritten
  – *Study Instance UID:* Overwritten
  – *Series Instance UID:* Overwritten
- *Station Name:* Overwritten as CONFIDENTIAL
- *Source Application Entity Title:* Overwritten as CONFIDENTIAL
- The following IDs have been overwritten:
  – *Patient ID:* Overwritten as !T000 for all patients.
  – *Study ID:* Overwritten as CONFIDENTIAL
  – *Scheduled Procedure Step ID:* Overwritten as CONFIDENTIAL
- Sensitive numbers:
  – *Accession Number:* Overwritten as CONFIDENTIAL
- Obvious Patient information attributes:
  – *Patient’s Name:* Overwritten as CONFIDENTIAL
  – *Patient’s Birth Date:* Cleared
  – *Patient’s Sex:* Cleared
  – *Patient’s Age:* Cleared
- Dates and Times:
  – *Study Date:* Overwritten with a random date
  – *Series Date:* Overwritten with a random date
  – *Acquisition Date:* Overwritten with a random date
  – *Content Date:* Overwritten with a random date
  – *Acquisition DateTime:* Overwritten with a random date and time
  – *Study Time:* Overwritten with a random time
  – *Series Time:* Overwritten with a random time
  – *Acquisition Time:* Overwritten with a random time
  – *Content Time:* Overwritten with a random time

It is interesting to note that all of the Instance UIDs have been de-identified; as opposed to UID classes, which are typically used to dictate transmission protocols between different entities over a PACS network, and do not disclose any patient identity attributes. In Appendix A, a sample DICOM file, before and after the de-identification process at the source is shown for comparison.

Certain attributes, which require de-identification are not de-identified by the first stage of de-identification at the source. In the next section, these attributes are described and the second stage of the de-identification process is explained.

### 4.2. Stage 2: Validation and Further De-Identification of DICOM

After several workshops with clinical experts that interact with NIMIS, the DICOM attributes that require further de-identification after stage 1 have been established. These workshops used sample data that were already partially de-identified by the export process at the source. Appendix B shows a list of DICOM attributes and indicates their status after stage 1. After having discussions with an expert radiologist and investigating each DICOM attribute in detail, the keyword SAFE is assigned to the attribute, which means that the attribute can be left alone, whereas NOT SAFE means the attribute needs to be de-identified in stage 2 of the process (Appendix B). The learning from the workshop along with the exhaustive attribute-level exploration are put in the form of an open-source automated de-identification tool [36].

The following attributes are those de-identified in stage 2 of the process:

- Private attributes:
  – *Private Creator:* Removed
  – *[Anonymization Status]:* Removed
- Sequential attributes:
  – *Request Attributes Sequence:* Removed because one of its sub-attributes (Requested Procedure ID) is determined as NOT SAFE by the clinical experts. It would be worthwhile to note that the reason that the Requested Procedure ID attribute is not directly removed is that it is a Type 1C attribute, whose existence is conditioned over the existence of the Request Attributes Sequence, which is a Type 3 attribute and can be safely removed.
- Dates and Times:
  – *Date of Last Calibration:* Overwritten with a random date
  – *Time of Last Calibration:* Overwritten with a random time
- Names:
  – *Institution Name:* Cleared
  – *Institution Address:* Cleared
  – *Referring Physician’s Name:* Cleared
- Comments:
  – *Image Comments:* Cleared
- UIDs:
  – *Irradiation Event UID:* Removed
  – *Private Information Creator UID* (and Private Information as a result of this): Removed

After these attributes have been successfully de-identified, an additional step is taken to extract the values of patient sex and age range from the folder names. These can then be safely inserted into the now-de-identified DICOM header. Particular care is taken to ensure the patient’s age, as agreed with the clinical experts, and is applied to the correct age range, e.g., 25–30, the upper-bound value of 30 is inserted into the actual DICOM header. Finally, the folder names and directories will remain intact.

The final step is a visual confirmation that the DICOM images within the dataset do not contain any burned-in annotations and do not require any further image processing operations for de-identification.

## 5. Discussion

The process of DICOM header de-identification and data curation is expensive, time-consuming and demands significant computing and human resources. The manual de-identification process is prone to human errors and fatigue. Automated DICOM de-identification tools serve as an alternative solution to manual and labour-intensive processes but require evaluation before they can be deployed in real-world applications. Some of the important considerations for evaluating the DICOM de-identification tools and processes are as follows [35]:

- It is advisable and often required to de-identify data at the source site (e.g., the hospital where the data are generated) before moving it off-site.
- Apart from DICOM header de-identification, there is a need for handling and validating the de-identification of burned-in pixel annotations as well as the facial features, where possible. In particular, it should be noted that in certain applications of Artificial Intelligence (AI) in radiation therapy, radiography and head and neck scans, the facial feature information might be necessary. For the current dataset, it has been confirmed that there are no facial features recorded in the DICOM images.
- There is a need to actively evaluate the tools’ compliance with DICOM standards by validating conformance to DICOM Application-Level Confidentiality Profile Attributes (ALCPA) (DICOM Application-Level Confidentiality Profile Attributes: https://dicom.nema.org/dicom/2013/output/chtml/part15/chapter_E.html#table_E.1-1, accessed on: 4 April 2022.).
- One should define concrete privacy preservation requirements for the specific use case and data at hand.
- One should ensure traceability and compliance audit by keeping a record of software, version, affected data portion, results, etc. for every de-identification event.

Although, the existing DICOM de-identification tools de-identify the PII attributes in the DICOM header, they do not provide a standard approach for patient privacy preservation. The proposed two-stage DICOM header de-identification process adopts an exhaustive attribute-level exploration strategy to identify the PII. Both the de-identification method and its details have been developed in collaboration with expert radiologists and DICOM data model experts and serve as potential best practices for DICOM header de-identification. Furthermore, the developed DICOM-compatible de-identification tool is standalone and can be easily integrated with the modern PACS and/or cloud computing infrastructures.

The minimum expected requirement from any DICOM de-identifier tool is to protect the PII of the patients. In the proposed de-identification approach of this paper, there are a number of criteria that enhance the credibility and effectiveness of a DICOM de-identification process (given in Table 1); which have been addressed and alleviated. These are explained below:

- *Fine-grained de-identification details*: It is essential for the credibility of any DICOM de-identification tools to provide a detailed explanation of the employed methods for DICOM de-identification. However, the existing literature lacks fine-grained de-identification details, which makes it difficult for the readers to elicit the best practices for de-identifying DICOM files. In this paper, an exhaustive attribute-level two-stage DICOM de-identification process has been described in a step by step fashion. In addition, the best practices of DICOM header de-identification have been described in order to ensure compatibility with the DICOM standard. The considerations outlined for DICOM de-identification in this paper such as the importance of considering *attribute type* and *value representation* will provide a guideline for clinical experts and researchers towards developing tools that ensure patients’ privacy.
- *Conforming to the DICOM standard*: Although most of the existing techniques claim to comply with the DICOM standard, the lack of sufficient details regarding their proposed de-identification methods, makes it difficult to ensure their DICOM standard compatibility. As a result, one can only hope that these approaches will abide by the DICOM standard and can be safely deployed in a DICOM-driven PACS network. However, this work has provided a highly detailed de-identification process in order to ensure the reader that the proposed method fully conforms to the DICOM standard and is deployable in a PACS network.
- *Validation with expert radiologists*: It is essential to consult and validate the de-identification of PII in DICOM file headers with expert clinical radiologists to ensure that the de-identification process will not jeopardise the diagnosis process by the radiologists. The validation of the de-identification methods by a domain expert can further guarantee the deployability of such methods in a real-life production environment. During the development of the proposed two-stage de-identification approach, multiple workshops have been held with expert radiologists to validate DICOM compatibility and as well as the integrity of the de-identified DICOM files. To the best of our knowledge, the state-of-the-art de-identification approaches do not incorporate this step. In the future work, it is hoped that consultation and validation with domain experts will become common practice in the future, especially in medical applications where the proposed methods have a direct impact on patient’s well-being.
- *Open source*: In order to ensure reproducibility, it is crucial to always share the underlying code for the developed methods, and this becomes even more important in medical applications. The de-identification tool developed in this paper is open-source and publicly available on [36].
- *Deployment in production environments*: Not all of the de-identification tools have been developed and tested by deploying them in an actual production environment. The proposed method has now been rigorously tested and fully deployed in the Medical Imaging Ireland (Med-I) platform.

During the constant effort to keep the two-stage DICOM de-identification method compatible with the DICOM standard, we have learned the following lessons:

- When it comes to DICOM de-identification, one must determine whether de-identifying the DICOM header attributes would suffice or a pixel-level de-identification is also required.
- Upon de-identifying any DICOM header attributes, one should constantly prevent violating the DICOM standard by checking the (1) *type* and (2) *value representation* of the attributes to be de-identified. Any violation could result in DICOM incompatibility and potential issues in a PACS network during the deployment phase.
- When developing DICOM de-identification tools, one should always be aware of the fact that, depending on the scanner machine, different DICOM attributes might be used in the generated DICOM files. As a result, it is a good practice to ensure that the developed de-identification method is both standalone and independent of the imaging modality in order to ensure the generalisability of these methods across multiple machines.

As future work, the following will be addressed: (1) Add the option of choosing the de-identification strategy for each attribute of interest, to the developed de-identification tool. For a given attribute, the de-identification strategies include replacing its value, randomising its value, removing the entire attribute, and clearing its value to an empty string. These strategies are tightly related to the attribute type and the value representation (VR) of each DICOM attribute. (2) Explore more medical imaging data from other sources and increase the robustness and scalability of the de-identification systems.

## 6. Conclusions

This paper reviewed the major challenges for de-identification in medical image data and presented a systematic approach for the preservation of patients privacy using the de-identification of PII in DICOM based medical imaging datasets. In this paper, a two-stage process of de-identification of CT scan images is proposed and developed, using a DICOM-compatible and verifiable approach while detailing the attribute-based de-identification decision, where the attribute type and Value Representation (VR) play an important role to ensure DICOM compatibility after de-identification. The first stage of the de-identification process involved the substitution and removal of some of the PII attributes in the DICOM headers at the source when exported from the PACS. The second stage performed a detailed attribute-level investigation of the DICOM headers and identified the PII revealing attributes that needed further de-identificatation, while respecting the compatibility with the DICOM standard. We further provided essential requirements towards enhancing the credibility and effectiveness of DICOM de-identification tools and evaluated the proposed approach in comparison with the state-of-the-art.

## Figures and Tables

**Table 1:** Comparative analysis of DICOM de-identification methods and tools.

Papers	Fine-Grained De-Identification Details	Conforming to DICOM Standards	Validation with Expert Radiologists	Open Source	Deployment in Production Environments
[15]		🗸		🗸	🗸
[13]		🗸		🗸	🗸
[17]		🗸			
[18]		🗸			
[19]		🗸		🗸	
Proposed methods	🗸	🗸	🗸	🗸	🗸

**Figure A1:**
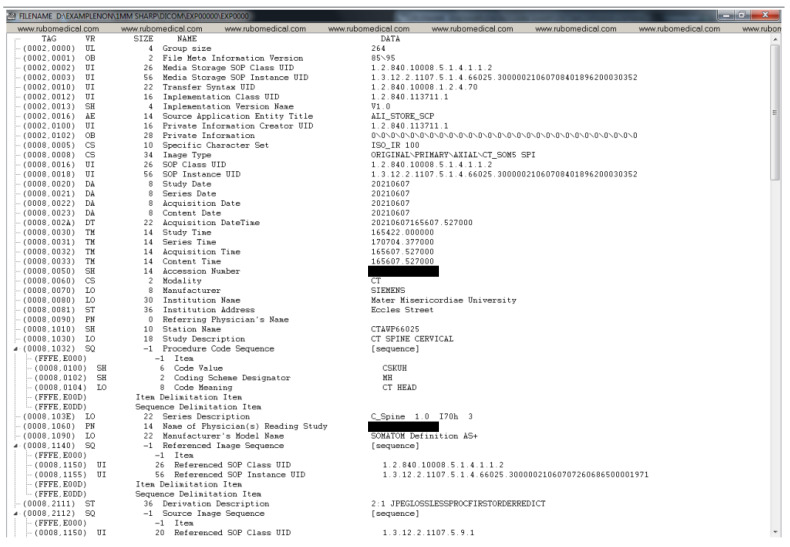
DICOM header data before de-identification (part 1).

**Figure A2:**
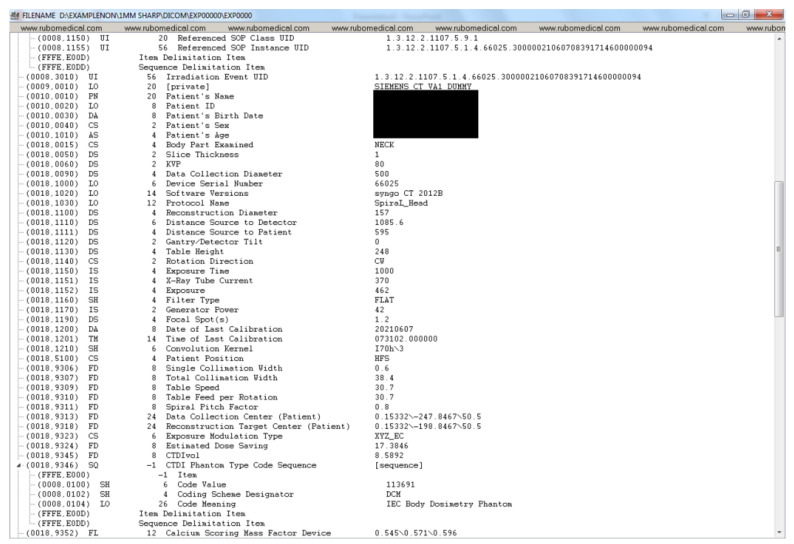
DICOM header data before de-identification (part 2).

**Figure A3:**
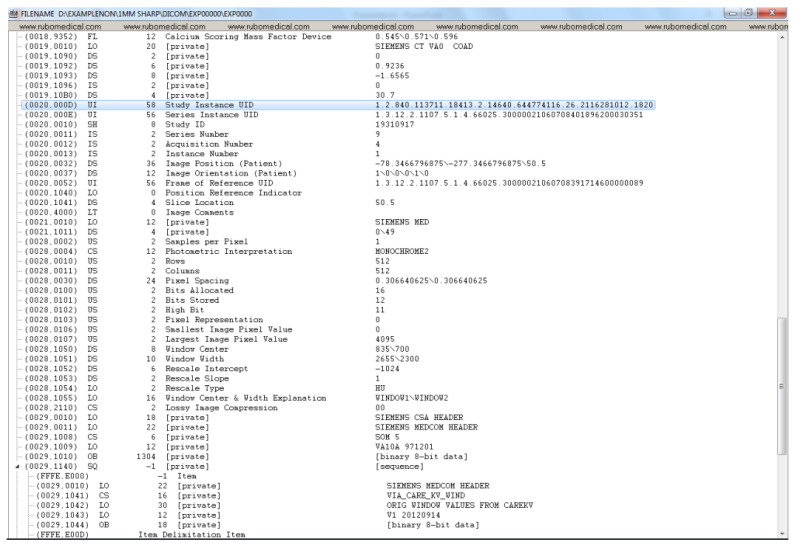
DICOM header data before de-identification (part 3).

**Figure A4:**
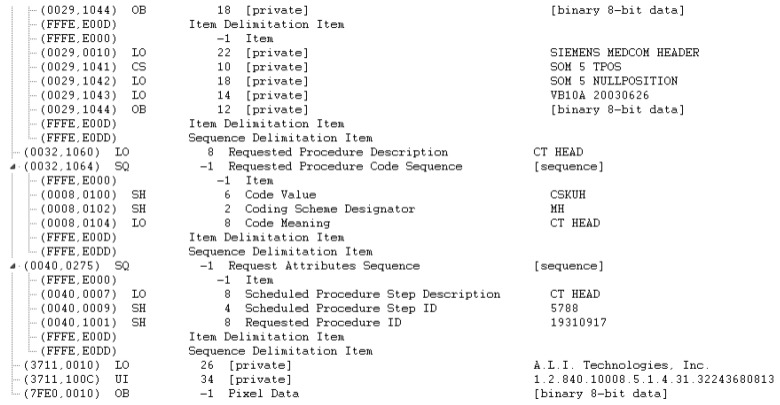
DICOM header data before de-identification (part 4).

**Figure A5:**
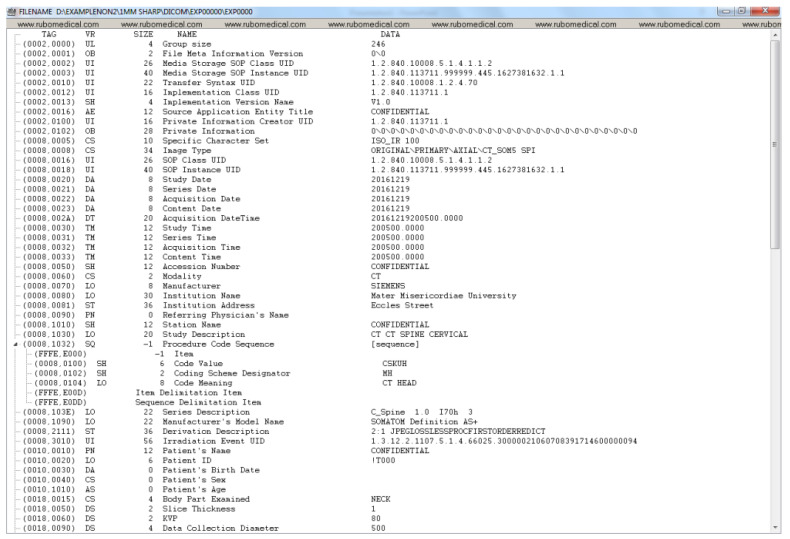
De-identified DICOM header data (part 1).

**Figure A6:**
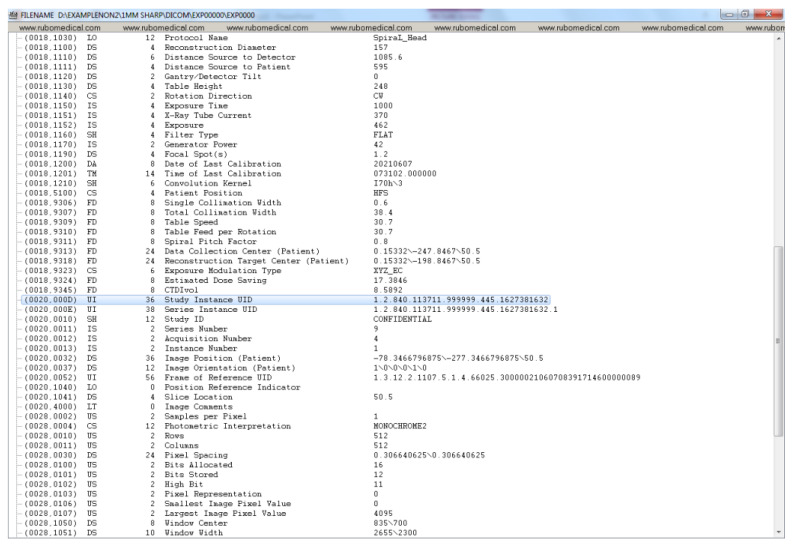
De-identified DICOM header data (part 2).

**Figure A7:**
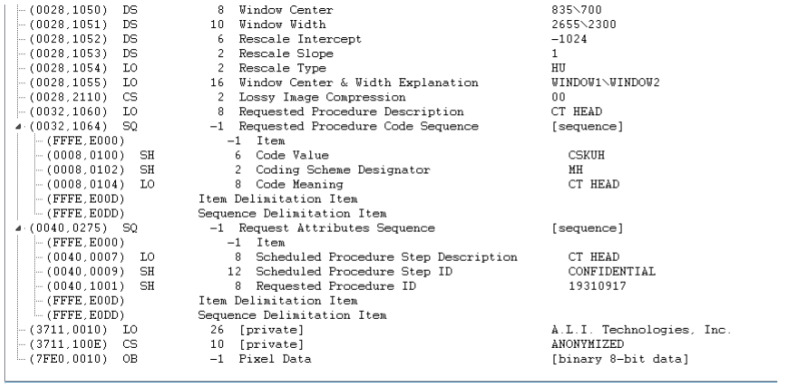
De-identified DICOM header data (part 3).

## Data Availability

Not applicable.

## Abbreviations

The following abbreviations are used in this manuscript:

ML	Machine Learning
AI	Artificial Intelligence
ALCPA	Application-Level Confidentiality Profile Attributes
HSE	Health Service Executive
PACS	Picture Archiving and Communication System
NIMIS	National Integrated Medical Imaging System
DICOM	Digital Imaging and Communications in Medicine
CT	Computed Tomography
PII	Personally Identifiable Information
PHI	Protected Health Information
GDPR	General Data Protection Regulation
UID	Unique Identification Number
NIfTI	Neuroimaging Informatics Technology Initiative
MINC	Medical Imaging NetCDF (Network Common Data Form)
MRI	Magnetic Resonance Imaging

## Appendix A. Example DICOM Header before and after De-Identification by the Clinical Team

Figure A1, Figure A2, Figure A3 and Figure A4 lists the DICOM headers for a patient case without any de-identification. The clearly visible PII such as patient name, age, and sex have been removed. Figure A5, Figure A6 and Figure A7 shows the same attribute after they pass through the stage 1 of the de-identification process.

## Appendix B. Detailed Examination of DICOM Headers from an Expert Radiologist Perspective

Table A1, Table A2 and Table A3, lists the outcomes of attribute-level investigation of DICOM headers from a radiologist point-of-view. The key word ‘de-identified by Export Process’ means that the DICOM attribute has been de-identified at the source (i.e., NIMIS), ‘SAFE’ means the attribute do not need any de-identification action, and ’NOT SAFE’ means the attribute needs further de-identification in stage 2.

**Table A1:** Detailed examination of DICOM headers (part 1).

Name	Value	De-Identification	Value Across	Value Across
		Status	All Patients	a Given Patient
File Meta Information Group Length	246	SAFE		
File Meta Information Version	b’\x00\x01’	SAFE		
Media Storage SOP Class UID	1.2.840.10008.5.1.4.1.1.2	SAFE	same	same
Media Storage SOP Instance UID	1.2.840.113711.999999.255.1620909105.1.1	SAFE	different	different
Transfer Syntax UID	1.2.840.10008.1.2.4.70	SAFE	same	same
Implementation Class UID	1.2.840.113711.1	SAFE	same	same
Implementation Version Name	V1.0	SAFE		
Source Application Entity Title	CONFIDENTIAL	de-identified by		
		Export Process		
Private Information Creator UID	1.2.840.113711.1	SAFE	same	same
Private Information	b’ALI DICOM OEM version 3.7.21’	SAFE		
Specific Character Set	ISO_IR 100	SAFE		
Image Type	ORIGINAL; PRIMARY;	SAFE		
	AXIAL; CT_SOM5 SPI			
SOP Class UID	1.2.840.10008.5.1.4.1.1.2	SAFE	same	same
SOP Instance UID	1.2.840.113711.999999.255.1620909105.1.1	SAFE	different	different
Series Time	164,557	RANDOMIZED by		
		Export Process		
Acquisition Time	164,557	RANDOMIZED by		
		Export Process		
Content Time	164,557	RANDOMIZED by		
		Export Process		
Accession Number	CONFIDENTIAL	de-identified by		
		Export Process		
Modality	CT	SAFE		
Manufacturer	SIEMENS	SAFE		
Institution Name	Mater Misericordiae University	NOT SAFE		
Institution Address	Eccles Street Hospital Dublin IE	NOT SAFE		
Referring Physician’s Name		de-identified by		
		Export Process		
Station Name	CONFIDENTIAL	de-identified by		
		Export Process		
Name	Value	de-identification	Value Across	Value Across
		Status	All Patients	a Given Patient
Study Description	CT CT SPINE CERVICAL	SAFE		
Procedure Code Sequence	Code Value: CSKUH—Coding	NOT SAFE	different	
	Scheme Designator: MH—Code		same	
	Meaning: CT HEAD			
Series Description	C_Spine 1.0 I30s 3	SAFE		
Manufacturer’s Model Name	SOMATOM Definition AS+	SAFE		
Derivation Description	2:1 JPEGLOSSLESS	SAFE		
	PROCFIRSTORDERREDICT			

**Table A2:** Detailed examination of DICOM header (part 2).

Irradiation Event UID	1.3.12.2.1107.5.1.4.66025.	NOT SAFE	different	same
	3000002105071355105980			
Patient’s Name	CONFIDENTIAL	de-identified by Export Process		
Patient ID	!T000	de-identified by Export Process		
Patient’s Birth Date		de-identified by Export Process		
Patient’s Sex		de-identified by Export Process		
Patient’s Age		de-identified by Export Process		
Body Part Examined	NECK	SAFE		
Slice Thickness	1	SAFE		
KVP	100	SAFE		
Data Collection Diameter	500	SAFE		
Device Serial Number	CONFIDENTIAL	de-identified by Export Process		
Software Versions	syngo CT 2012B	SAFE		
Protocol Name	SpiraL_Head	SAFE		
Reconstruction Diameter	146	SAFE		
Distance Source to Detector	1085.6	SAFE		
Distance Source to Patient	595	SAFE		
Gantry/Detector Tilt	0	SAFE		
Table Height	193.5	SAFE		
Rotation Direction	CW	SAFE		
Exposure Time	1000	SAFE		
X-Ray Tube Current	198	SAFE		
Exposure	247	SAFE		
Filter Type	FLAT	SAFE		
Generator Power	32	SAFE		
Focal Spot(s)	1.2	SAFE		
Name	Value	de-identification Status	Value Across All Patients	Value Across a Given Patient
Single Collimation Width	0.6	SAFE		
Total Collimation Width	38.4	SAFE		
Table Speed	30.7	SAFE		
Table Feed per Rotation	30.7	SAFE		
Spiral Pitch Factor	0.8	SAFE		
Data Collection Center (Patient)	0.142578125;	SAFE		
	−193.357421875; −569.4			
Reconstruction Target Center (Patient)	37.142578125;	SAFE		
	−144.357421875; −569.4			
Exposure Modulation Type	XYZ_EC	SAFE		
Estimated Dose Saving	43.1912	SAFE		
CTDIvol	9.747946319	SAFE		
Study Instance UID	1.2.840.113711.999999.	NOT SAFE	different	same
	255.1620909105			
Series Instance UID	1.2.840.113711.999999.	NOT SAFE	different	same
	255.1620909105.1			
Study ID	CONFIDENTIAL	de-identified by Export Process	same	same
Series Number	10	SAFE		
Acquisition Number	4	SAFE		
Instance Number	1	SAFE		
Image Position (Patient)	−35.857421875;	SAFE		
	−217.357421875; −569.4			

**Table A3:** Detailed examination of DICOM header (part 3).

Image Orientation (Patient)	1; 0; 0; 0; 1; 0	SAFE		
Frame of Reference UID	1.3.12.2.1107.5.1.4.66025.	SAFE	different	same
	3000002105071355105980			
Position Reference Indicator		SAFE		
Slice Location	−569.4	SAFE		
Image Comments		NOT SAFE		
Samples per Pixel	1	SAFE		
Photometric Interpretation	MONOCHROME2	SAFE		
Rows	512	SAFE		
Columns	512	SAFE		
Pixel Spacing	0.28515625; 0.28515625	SAFE		
Bits Allocated	16	SAFE		
Bits Stored	12	SAFE		
High Bit	11	SAFE		
Pixel Representation	0	SAFE		
Smallest Image Pixel Value	0	SAFE		
Largest Image Pixel Value	2921	SAFE		
Window Center	60; 50	SAFE		
Window Width	420; 400	SAFE		
Name	Value	de-identification Status	Value Across	Value Across
			All Patients	a Given Patient
Rescale Intercept	−1024	SAFE		
Rescale Slope	1	SAFE		
Rescale Type	HU	SAFE		
Window Center & Width Explanation	WINDOW1; WINDOW2	SAFE		
Lossy Image Compression	0	SAFE		
Requested Procedure Description	CT HEAD	SAFE		
Requested Procedure Code Sequence	Code Value: CSKUH—Coding	NOT SAFE	different same	
	Scheme Designator: MH—Code			
	Meaning: CT HEAD			
Request Attributes Sequence	Scheduled Procedure Step Description:	NOT SAFE	same different	
	CT HEAD—Scheduled Procedure Step			
	ID: CONFIDENTIAL—Requested			
	Procedure ID: 19,930,118			
Date of Last Calibration	20,210,507	NOT SAFE		
Time of Last Calibration	120,556	NOT SAFE		
Convolution Kernel	I30s; 3	SAFE		
Patient Position	HFS	SAFE		
Study Date	20,170,822	RANDOMIZED by Export Process		
Series Date	20,170,822	RANDOMIZED by Export Process		
Acquisition Date	20,170,822	RANDOMIZED by Export Process		
Content Date	20,170,822	RANDOMIZED by Export Process		
Acquisition DateTime	2.02 × 10${}^{13}$	RANDOMIZED by Export Process		
Study Time	164,557	RANDOMIZED by Export Process		
